# Making space for Aboriginal and Torres Strait Islander community health workers in health promotion

**DOI:** 10.1093/heapro/daz035

**Published:** 2019-06-01

**Authors:** Kathleen P Conte, Josephine Gwynn, Nicole Turner, Claudia Koller, Karen E Gillham

**Affiliations:** 1 University Centre for Rural Health and the Menzies Centre for Health Policy, School of Public Health, Faculty of Medicine and Health, University of Sydney, Level 2, Charles Perkins Centre, University of Sydney, NSW 2006, Australia; 2 Faculty of Health Sciences and the Charles Perkins Centre, University of Sydney, Sydney, Australia; 3 University of Canberra, Canberra, Australia; 4 Rural Doctors Network, New South Wales, Australia; 5 Faculty of Health and Medicine, University of Newcastle, Newcastle, Australia; 6 Population Health, Hunter New England Local Health District, Wallsend, Australia

**Keywords:** community health promotion, indigenous, empowerment, participation, Aboriginal health

## Abstract

Despite a clear need, ‘closing the gap’ in health disparities for Aboriginal and Torres Strait Islander communities (hereafter, respectfully referred to as Aboriginal) continues to be challenging for western health care systems. Globally, community health workers (CHWs) have proven effective in empowering communities and improving culturally appropriate health services. The global literature on CHWs reflects a lack of differentiation between the types of roles these workers carry out. This in turn impedes evidence syntheses informing how different roles contribute to improving health outcomes. Indigenous CHW roles in Australia are largely operationalized by Aboriginal Health Workers (AHWs)—a role situated primarily within the clinical health system. In this commentary, we consider whether the focus on creating professional AHW roles, although important, has taken attention away from the benefits of other types of CHW roles particularly in community-based health promotion. We draw on the global literature to illustrate the need for an Aboriginal CHW role in health promotion; one that is distinct from, but complementary to, that of AHWs in clinical settings. We provide examples of barriers encountered in developing such a role based on our experiences of employing Aboriginal health promoters to deliver evidence-based programmes in rural and remote communities. We aim to draw attention to the systemic and institutional barriers that persist in denying innovative employment and engagement opportunities for Aboriginal people in health.

*‘Too many white Australians think the door opens to opportunity from the outside, when you’ve got to be let into the door from the inside’.* Noel Pearson, Aboriginal activist, as quoted in *The Australian*, 7 May 2015. (Bita, 2015)

## INTRODUCTION

Aboriginal and Torres Strait Islander cultures in Australia are acknowledged to be the oldest living cultures in the world (Australian Government, 2017a), maintaining thriving and diverse communities for over more than 60 000 years, and implementing land management practices that are exemplary in their sustainability and productivity (Pascoe, 2018). Hereafter, we use the term Aboriginal to describe the many different clans that make up this diverse peoples, including those from the Torres Strait. Following the British invasion and subsequent colonization of Australia, Aboriginal people across the nation suffered a sudden and complete rupture to all aspects of life including kinship, language, spirituality and culture. The resulting health disparities experienced by Aboriginal people since colonization, and the inequalities that contribute to them, are well documented (AIHW, 2015). Despite the preponderance of evidence as to these inequities there has been only marginal progress in implementing effective strategies to improve health (McCalman *et al.*, 2016). Not enough research has focused on how Aboriginal knowledge is reflected in health programmes and services, and there are continued calls for Aboriginal people to be leaders of health-promoting endeavours (National Congress of Australia’s First People, 2016; NHMRC, 2018). However, combatting systemic racism and reorienting the institutions of the dominant non-Aboriginal culture—i.e. government, health care, education—to include Aboriginal people in decision making and to enable their leadership is proving to be an ongoing challenge in both global and local health settings (George *et al.*, 2015). The opening quote of this paper draws attention to this often-contested issue.

Community ownership of decision making for health has long been recognized as key to addressing the social determinants of health that underlie health disparities (WHO, 1978). Internationally, community health workers (CHWs) enable community involvement in health systems—particularly among minority communities—and contribute to positive health outcomes in a variety of settings (Goris *et al.*, 2013; Kim *et al.*, 2016). In the USA, for example, the Indian Health Service has funded American Indian ‘Community Health Representatives’ since 1968 (Satterfield *et al.*, 2002). These health workers provide links between communities and health services, and build trust, relationships and culturally appropriate education and care. Maori CHWs play a similar bridging role in New Zealand by linking community members with health interventions and clinical services, providing health education and also working alongside traditional healers and supporting tribal development (Boulton *et al.*, 2009).

In Australia, CHWs are largely operationalized as Aboriginal Health Workers (AHWs), although there is considerable variation in the kinds of roles they perform. The result is that some AHWs experience inflated role expectations that can contribute to unmanageable workloads and stress, reduced job satisfaction, and barriers to integration with other members of the health workforce (Bailie *et al.*, 2013; Schmidt *et al.*, 2016). Yet variations in role definition for CHWs, and the associated problems, are not unique to Australia (Topp *et al.*, 2018) and are well documented in the broader global CHW literature (Olaniran *et al.*, 2017; Taylor *et al.*, 2017). This variation is problematic as it impedes research into how CHWs influence health outcomes.

In this paper, we explore the lack of differentiation in the global literature between the types of CHW roles both internationally and within the Australian context. Differentiating the various types of CHW roles has enabled us to articulate the need for a specific community health promotion role, one that is distinct from, but complementary to, that of AHWs in clinical settings. The impetus for writing this paper came from the experiences of two of the authors (NT and JG), an Aboriginal and a non-Aboriginal woman, who have worked in partnership for more than 15 years delivering and evaluating health promotion programmes in Australia. The challenges we experienced in creating Aboriginal CHW-type positions within two mainstream health promotion programmes caused us to question whether the focus on AHW roles had created unintended barriers to involving Aboriginal people in other opportunities to address health. By detailing our experience in creating community-based, Aboriginal CHW positions in health promotion, we aim to draw attention to the systemic and institutional barriers that impede expanding employment opportunities for Aboriginal people wanting to work in health.

## CHWs AND AHWs

Broadly, CHWs are individuals who may or may not be paid, who work towards improving health in their assigned communities and who often share some of the qualities of the people they serve. These may include similar cultural, linguistic or demographic characteristics; health conditions or needs; shared experiences or simply living in the same area. However, the degree to which CHWs demographic or experiential profiles ‘match’ the target population also varies. And while most bring cultural and community knowledge to the role, many CHWs have little or no training in Western medicine or in navigating its health systems prior to becoming CHWs (Olaniran *et al.*, 2017).

There is less agreement on the specifics of the CHW role including what they do, how they are trained, how these parameters link to outcomes, and even the titles they are given. One review evidenced 120 terms used to describe CHW roles including variants of ‘lay health educators’, ‘community health representatives’, ‘peer advisors’ and ‘multicultural health workers’ (Taylor *et al.*, 2017). Syntheses of literature on CHWs illustrate that the tasks they undertake are highly varied but often inadequately or inconsistently defined (Jaskiewicz and Tulenko, 2012; Kim *et al.*, 2016). These issues, coupled with a general lack of contextual information about the role of CHWs, make it difficult to determine patterns or predictors of success. This lack of clarity is documented as an ongoing barrier to the sustainability of CHW programmes, sometimes causing negative impacts on the workers themselves including burnout due a lack of appropriate training and mentoring support (Jaskiewicz and Tulenko, 2012; Schmidt *et al.*, 2016). One review concluded that ‘the [CHW] role can be doomed by overly high expectations, lack of clear focus, and lack of documentation’ [(Swider, 2002), p. 19].

Previous research has classified CHW roles into typologies of main tasks and activities performed (Olaniran *et al.*, 2017; Taylor *et al.*, 2017). These include providing: (i) social support, (ii) clinical care, (iii) service development and linkages, (iv) health education and promotion, (v) community development, (vi) data collection and research and (vii) activism. In practice, CHW activities overlap substantially, and tasks regularly extend across categories—both formally and informally (Jaskiewicz and Tulenko, 2012). In Table 1, we present different CHW role types alongside the theoretical models that underpin each. Linking roles to theory can help differentiate and specify the mechanisms by which CHWs are meant to influence health through the core tasks they perform, and the specific skills related to each task.

**Table 1:** Categorization of types of CHW roles

Category and description	Key theorists or models that are useful for theorizing CHW roles	Core task and skills	Related terms	Examples of CHW role operationalized in the literature
Activist/change agent Takes part in or leads activities to create social or political change. Creates awareness for and mobilizes community for change	Social change, community organizing and empowerment theorists Community Organizing: Alinsky (2010) and Minkler and Wallerstein (2012) Social change: Freire (2000)	Activism and community development Problem-solving Community organizing and development Systems thinking	Community organizers, community health leaders, community development worker	Women’s Health Leadership Institute trains local CHWs to influence local community change that targets health disparities in women’s health (Ingram *et al.*, 2016)
Advocate Supports and promotes the rights of individuals, families or communities	Political science theories Citizen Advocacy Model: Wolf Wolfensberger [as cited in Health Consumers Queensland (2011)] Coalition theory: Sabatier (1986) Power politics: Mills (2000)	Activism and social support (informational, instrumental, appraisal and emotional types) Knowledge of health care or institutional system Systems thinking and problem-solving	Patient advocates, ombudsmen, patient representatives, health care advocate, bilingual health advocate	The Wishard Volunteer Advocates Programme matches trained volunteers with unbefriended, incapacitated individuals for whom they facilitate health care decision-making, participate in guardianship hearings, and help ensure provision of clothes and personal items (Bandy *et al.*, 2014)
Advisor/coach Provides tailored education and support to a client to achieve a goal or to do a particular activity	Socio-behavioural and stage theorists Health Belief Model: Hochbaum (1958) Social Cognitive Theory: Bandura (1997) Theory of Planned Behaviour and Reasoned Action: Fishbein and Ajzen (1975) Transtheoretical model of change: Prochaska and Velicer (1997)	Education and social support (informational and appraisal types) Communication Expertise in content area	Labour coaches, mentors, lay health advisors, mentors	Peer health coaches provided targeted support via in-person and telephone meetings, at least twice a month for 6 months to individuals with poorly controlled diabetes. Coaches helped design action plans, provide support and follow-up to progress on achieving patient-identified goals (Thom *et al.*, 2013)
Researcher Conducts or participates in locally-based data collection to identify, monitor and research problems	Community-based participatory research theorists Israel *et al.* (1998) See also activist/change theorists above	Research activities Data collection, analysis or interpretation skills	Health surveillance assistants	Marshallese community members in Arkansas, USA were employed as research coordinators to collect data and participate in interpretation to identify needs and improve community health (Purvis *et al.*, 2017)
Companion Provides time, support, assistance and/or friendship. Unlike coaches, companions may provide no tangible assistance other than just being present	Social network and social support theorists House (1981)	Social support (informational, instrumental, appraisal and emotional types) Willingness and ability to spend time with and provide assistance, caring or information to another person	Senior companions, social supporter, peer supporter, volunteer befriending	The Senior Companion Programme trains then matches volunteers >60 years old with older adults with unmet assistance needs living in the community. Volunteers provide a range of social support including companionship, rides to appointments and errands and other tasks (Butler, 2006)
Health aide, or health worker Provides basic health services	*Bio-medical and psycho-social models* of health and illness that assume causal relationships between illness and disease, and the delivery of medical interventions to intervene See, for example, Wade and Halligan (2004)	Clinical care and education Administer screening tests, document vital statistics, distribute medications, conduct basic procedures and/or provide health information	CHW, allied health personnel, health aides, nursing assistants	In Malawi, CHWs called ‘health surveillance assistants’ provide in-home monitoring of adherence to and dispensing of medications (Celletti *et al.*, 2010)
Health educator Provides information and teaches skill development	Socio-ecological model of health Bronfenbrenner (1992) See also socio-behaviour and change theorists above	Education Knowledgeable in specific content area Communication and teaching skills	Health education worker, health trainers, peer educator	The Strong Women programme uses a train-the-trainer model to train lay community members to be programme leaders. Trained lay leaders teach and lead exercise classes in their local communities (Washburn *et al.*, 2014)
Navigator/case manager Helps clients organize and coordinate services. In doing so, may provide education, help organize and attend doctor’s visits, facilitate access and accompany patients	Combines medical models with socio-behavioural theories to improve utilization of care services	Service development and social support (instrumental and informational types) Knowledge of particular institution or (health) system Ability to problem-solve Communication	Lay patient navigator; care facilitator; health surveillance assistant	In low- and middle-income countries, CHWs provide case management of childhood illness (e.g. malaria, diarrhoea, acute respiratory infections and measles) including distributing medications, providing assessments and making referrals (Gilroy and Winch, 2006)
Outreach worker Identifies, engages and creates linkages between underserved population and an organization	Opinion leader models Katz and Lazarsfeld (2017)	Service development Assess needs of people and communities Collect data to document needs Make referrals	Peer outreach workers, street outreach workers, link workers	Tribal Veteran Representatives are Native Veterans trained as CHWs who identify unenrolled American Indian and Alaskan Native veterans and assist them in enrolling in Veterans Health Administration system for health care services (Kaufmann *et al.*, 2014)
Popular opinion leader/role model Recognizable leader in community or social group that can influence behaviours and culture change	Diffusion of innovations theory and opinion leader models Rogers (2003) Katz and Lazarsfeld (2017)	Community development and Social support (informational) Model risk-reduction attitudes and behaviours Deliver effective health messages	Peer educators, peer informants, natural helpers, role models, champions	Key leaders in MSM communities are identified, recruited and trained to disseminate and model HIV risk reduction messages (NIMH Collaborative HIV/STD Prevention Trial Group, 2007)

As Avery and Fernandez (Avery and Fernandez, 2012) pertinently discuss, the terms by which CHWs are classified are not simply semantics; they carry important implications about the work performed, the training needs for each role, how CHWs should be recruited for particular roles, and how their success will be measured. A ‘companion’, for example, could provide social support and friendship to an infirm neighbour simply by drawing on shared cultural values or experiences, so may or may not require training and/or affiliation with an organized programme. Conversely, because a ‘health worker’ role usually requires delivery of clinical services and health education within a Western health care system, training will be needed to ensure an appropriate level of health care is provided. By linking the tasks that CHWs perform in their ‘role’ to underlying theories of change for how this work is meant to impact on health, we aim to illustrate key differences in role types that enable distinctions as to how CHW roles meaningfully differ from one another.

Like CHWs internationally, there is considerable variation in how Australian AHW roles are defined and the contexts in which they work. Aboriginal people have a long history of working in health that pre-dates colonialization, and recently the important role that traditional healers play is becoming more recognized and integrated into Western clinical systems (Australian Government, 2017b). The ‘AHW’ role was first established in the Northern Territory and recognized by the Western health system in the 1950s (Topp *et al.*, 2018). It was formally incorporated into Australia’s national health system in 2008 (National Aboriginal and Torres Strait Islander Health Worker Association, 2016). Individuals can become an AHW if they are pursuing or hold a Certificate III, IV or higher degree diploma in, for example, primary health care, public health or a specific area of practice such as mental health. In the mainstream health care sector, AHWs serve in ‘health worker’ or ‘outreach’ roles, providing clinical services, community outreach and education to improve access, health outcomes and the cultural appropriateness of services (McDermott *et al.*, 2015). Some also have specified AHW positions in prevention and health promotion. But the delivery of Indigenous health promotion in Australia is best exemplified by the work of Aboriginal Community Controlled Health Organisations (ACCHOs).

ACCHOs are primary health care services operated by the local Aboriginal community that they serve (NACCHO, 2018). Their approach to providing comprehensive and culturally competent services draws on the cultural knowledge, beliefs and practices of their communities, and aligns with the Ottawa Charter principles aimed at enabling communities to take control of their own health care needs (WHO, 1986). AHW positions within ACCHOs may, therefore, reflect the full range of role types outlined in Table 1.

It is primarily within ACCHO-developed community programmes that other types of CHW roles and models for their delivery have been implemented, for example, lay-leader or peer-to-peer education models (McPhail-Bell *et al.*, 2017). Yet many of these initiatives are only documented in programme reports within the ‘grey literature’ with much of the work undertaken in Aboriginal health promotion remaining under-researched and underreported (McCalman *et al.*, 2016). Studies that do report on AHW and community-led programmes frequently lack information about the role and definition of AHWs, the activities they perform, and their recruitment, training, compensation and supervision.

The lack of research and detailed description of both AHW and other CHW models makes it difficult to differentiate the various role types, which results in difficulty specifying their unique training needs, recruitment methods and how best to advocate for particular roles in different contexts. For example, in 2009 the Australian Government substantially invested in the Indigenous Chronic Disease Package a ‘multi-faceted strategy … which aim(ed) to enhance the capacity of the primary health care system around preventative health and effective chronic disease management’ [(KPMG, 2014), p. 8]. One of the three elements of the Package was workforce expansion and included an entry-level Aboriginal Outreach Worker role. The resulting evaluation, however, found this role was conceptualized differently across settings resulting in inflated role expectations and, therefore, problems in establishing appropriate training and supervision systems (Bailie *et al.*, 2013). The final report from KPMG recommended that:

Future programs on preventive health for Aboriginal and Torres Strait Islander people would benefit from undertaking a more in depth analysis of potential models and approaches that could be developed and used to both build and maximize the uptake of effective health promotion approaches in community settings using ground up approaches. (p55)

In the spirit of this recommendation, we now shift to describing our experience of implementing one such potential health promotion programme model in which we employed local Aboriginal community members. This experience came about from our 12 years of implementing two government-funded and prioritized healthy lifestyle behavioural and educational and research programmes to communities with large Aboriginal populations (Gwynn *et al*., 2014). Through this work, we identified a need for a CHW role distinct from that of an AHW given the contextual and theoretical factors underlying the content and settings in which the programmes were delivered. The activities of the CHW reflected both ‘educator’ and ‘change agent’ roles, a combination that exemplifies the aims of health promotion. Therefore, we refer to these CHWs hereafter as Community Health Promoters (CHPs).

## EMPLOYING ABORIGINAL CHPs

The two programmes we delivered aimed to promote wellness and reduce the prevalence of obesity and its associated chronic conditions (e.g. type-2 diabetes) among Aboriginal and non-Aboriginal school-aged children. The driving philosophy of both was to build local community capacity rather than exclusively produce better health outcomes. Therefore, in line with the National Health and Medical Research Council’s principles on Aboriginal and Torres Strait Islander research—i.e. respect, reciprocity, equity, survival and protection and responsibility (NHMRC, 2018)—we adopted the following commitments in both projects:

1. To employ mostly Aboriginal people from the participating communities
2. To employ those who were currently unemployed, including those with minimal work experience
3. To engage in inclusive programme delivery approaches by including Aboriginal and non-Aboriginal children in community services and programmes.

We identified that the role of CHPs was conceptually distinct to that of AHWs for several reasons. First, AHWs are often (but not always) employed through a medical service (e.g. an ACCHO), and the health promotion programmes they deliver may be perceived to be ‘health care’ by association. We wanted to employ people from the community who were clearly independent of the ‘health care’ context to reflect our philosophy of being community-based and to adhere to the community-delivery mechanisms underlying both programmes’ theory of change.

Second, we were committed to providing opportunities for members of the local community to gain employment and to improve their skill capacity without committing to formal training. Obtaining an AHW qualification is a substantive commitment and, therefore, a barrier for some, particularly where travel and time away from family and community are required to undertake the training. With high unemployment rates in many of the participating communities, we wanted to create opportunities for community members not already involved in health to gain job skills without the commitment and obstacles associated with undertaking a certification programme. Previous research (Kane *et al.*, 2016) also showed that the experience of having been a CHP increased an individual’s employment opportunities; improved their own, their families and their community’s knowledge and health literacy and has a consequent impact on the health behaviours of community members.

Finally, our primary goal was to recruit applicants who had strong cultural and community connections to ensure that both programmes were meaningful and of value to the communities involved. Therefore, cultural expertize and community connectivity were our primary recruitment criteria. As underscored by numerous studies (Goris *et al.*, 2013), we found that, with basic skills and commitments, most CHPs could acquire the content-specific knowledge and develop the skills relevant to deliver our programmes’ healthy lifestyle messages.

To date, we have recruited, employed and trained 41 Aboriginal CHPs to deliver two large-scale physical activity and nutrition programmes to school-aged children. One programme was part of a research endeavour in which CHPs collected evaluation survey data, encouraged physical activity through sports like ‘Traditional Indigenous Games’ and ‘Midnight Basketball’, and promoted better nutrition by establishing a community garden. It was delivered across two large regional communities over a 6-year period, with each community having its own team of up to six CHPs. The other is an ongoing 10-week programme delivered in schools and community organizations (sometimes more than once) across more than 25 communities of varying sizes. In each community, we employed up to three CHPs, with at least two recruited from the participating community or region. In both programmes, the CHPs worked an average of 10 h per week for 4–12 months and were supervised by a Senior Aboriginal Project Officer (SAPO and co-author) whose role we detail later in this paper.

CHPs ranged in age from 20 to 60 years, and just over 50% were women. We purposefully employed CHPs with a range of cultural backgrounds and experiences to maximize the diversity of Aboriginal people represented in leadership roles, and to provide equal employment opportunities. The primary requirements for employment were that CHPs came from the community in which they worked and shared its cultural characteristics; were committed and motivated to perform their work, be good role models, and energetic communicators. Community members were critical in identifying and recruiting potential CHPs through word-of-mouth referrals.

### Addressing barriers to creating Aboriginal CHP positions

Employing individuals with both limited employment experience and educational qualifications (some CHPs had not completed secondary school) was novel for the funding bodies of our programmes and presented challenges when setting up recruitment and hiring processes. With most government-funded health programmes preferencing the hiring of individuals with tertiary degrees in health, we had to be creative when carving out positions for CHPs and actively advocating for their continued employment and support. For example, we advocated for award wages being commensurate with local experience and qualifications rather than requiring institutionally recognized employment and education histories.

Navigating the bureaucratic administrative processes to recruit CHPs and execute their employment contracts was notably complicated because there were no established pathways for hiring local community members from a non-dominate culture who do not reflect the typical job candidate. Many candidates lacked the identification documents necessary for employment, and/or had no experience of the red tape and paperwork required to complete the employment process. For some, more usually Aboriginal men than women, convictions for prior and often minor offences committed at a young age further complicated the criminal background check, a requirement for such positions. We successfully addressed these barriers by working closely with the programme’s commissioning institution, conducting additional reference checks (in the case of prior convictions) and supporting CHPs to navigate, complete and submit paperwork for employment.

Once selected, the financial outlay required to navigate the employment process and fulfil its requirements can be formidable. Many applicants must travel from rural communities to regional centres where identification documents are issued and notarized and the employment processes conducted. In a traditional hiring model, costs for obtaining identification and proof of qualifications are incurred by applicants before they are hired, and thus before they can be reimbursed by wages from the position. For individuals under financial stress, such expenses can prohibit them from completing the employment process and illustrate how institutional barriers impede the employment of vulnerable people (Ferdinand *et al.*, 2014). In our two programmes, we assigned project funds to cover the costs associated with obtaining the paperwork required for the CHP application.

Once their employment status was finalized, CHPs faced additional challenges in completing employment-related processes. Access to the technology needed to do their job—i.e.submitting timesheets, accessing internet and email, having mobile phones with sufficient credit, etc.—was complicated because CHPs work and travel between rural and remote communities. This means that having a physical office space was often unfeasible (given expenses and lack of infrastructure), unnecessary (given the delivery of programmes is in school and community settings) and/or undesired by the CHPs who preferred to work from home given the distances involved.

Overcoming these barriers involved innovative and persistent action by senior project staff. For example, the SAPO worked closely with potential CHPs throughout the recruitment, training and employment process, providing technical assistance, navigating administrative procedures and solving technological issues. Most importantly, the SAPO gave applicants the emotional support and encouragement they needed to persist in working through the barriers.

When possible, CHPs obtained their training from the SAPO. This was not always achievable, however, particularly as one programme required all staff to complete 2 days of centralized training as part of the government administration of the programme. Targeted at upskilling programme deliverers from across the State, most of whom were health promotion professionals, this training was not designed to fit the needs of Aboriginal CHPs. Rather, it was reflective of ingrained, structural institutional practices that continue to pose barriers to the employment of Aboriginal CHPs. Many CHPs had never experienced formalized, structured training so, for them, attending the 2-day training in a major city was their first experience in navigating air travel, city transport and hotel accommodation.

As has been reported by other Aboriginal people (Gwynne and Lincoln, 2016), CHPs attending the training described feeling anxious about the quantity of information presented, unconfident and ‘shameful’ because of the gap in their knowledge and experience compared to the other professional attendees. To address these barriers, the SAPO attended all training sessions with the CHPs to provide support and ensure they felt safe to participate. Senior staff, including the SAPO and more experienced CHPs, also worked with new CHPs before and after training to establish expectations, build self-confidence and follow-up with additional information to assist them to integrate and apply content learning from the State training into their local settings.

In addition to supervising the CHPs, who worked together as a team to deliver programme content, the SAPO provided personal mentoring. Together with the more experienced CHPs, the team oriented new staff, explained and modeled role expectations. A key quality of the mentoring approach was to provide clear, focused, non-paternalistic and tailored direction to support the development of social and practical job skills among CHPs in a manner that reduces anxiety and shame and increases self-confidence. It also required the SAPO to be well connected to the CHPs for ongoing support and to answer questions when in the field, including being accessible after-hours for problem solving often via daily text messages.

### Benefits of the CHP model

The CHPs delivered programmes in school settings to both Aboriginal and non-Aboriginal children. This focus on inclusivity, in terms of both ethnicity and health risk factors, brought many advantages. It not only ensured inclusivity by spreading the message to everyone and creating common goals, but also reduced any stigma associated with targeting programmes only to at-risk groups. This is particularly important in small communities where people may feel singled-out if included or overlooked if excluded. In addition, it was beneficial to all children and parents to see an Aboriginal person as the main face of the programme. Aboriginal community members then see that education, physical activity and nutrition are culturally acceptable, while non-Aboriginal community members build relationships with CHPs thereby increasing their exposure to, and positive experiences with, Aboriginal people and culture. Such experiences are critical for improving cultural competency and facilitating reconciliation (Cinelli and Peralta, 2015). Finally, CHPs tailored messaging to make content culturally appropriate, thereby improving the relevance of the programme in the local context. These adaptations ensured that Aboriginal families felt accepted and able to participate more fully in the programme content.

As a result of this approach, programmes led by CHPs have enjoyed strong Aboriginal family participation and completion rates (Gwynn *et al*., 2014). Furthermore, the CHPs themselves have benefitted in several ways. Many who have not previously had positive experiences of the school environment found it empowering and reconciling to be successfully delivering health education in school settings. They also improved their skills, knowledge and confidence to interact with their communities, while gaining employment experience that will enable them to seek future work. Their families also benefitted from the CHPs applying their knowledge about nutrition and physical activity at home.

## DISCUSSION

Our experiences highlight the structural barriers that exist in employing Aboriginal people in a community-based CHP model, and illuminate the ethical implications of restricting employment opportunities to only one type of CHW role. The difficulties we encountered in setting up and running health promotion programmes delivered by CHPs illustrate how organizational practices, procedures, policies and infrastructure sustain racial inequalities (Ferdinand *et al.*, 2014; Schmidt *et al.*, 2016). A response to these factors requires new models of employment for all CHW roles, and programmes that are designed by Aboriginal people who then have ownership of their delivery. Although there is an emerging body of knowledge that addresses these factors, and Aboriginal leadership is repeatedly recommended, moving beyond aspiration into practice remains testing for mainstream organizations. For example, the lack of progress on key strategies such as Closing the Gap is indicative of the need for a new organizational framework of service delivery and employment. In Box 1, we offer considerations for developing community-based CHP roles.

AHWs also experience barriers similar to those described here, including difficulties in accessing training, lack of role clarity, poor recognition of their value and insufficient support (Gwynne and Lincoln, 2016; Schmidt *et al.*, 2016). The formalization of AHW positions through course accreditation has led to dedicated resources and infrastructure in an attempt to alleviate such barriers. Yet, it may have also further entrenched institutional racist practices by restricting Aboriginal and people in the health workforce to particular, defined and targeted roles. As a result, there is a growing impetus to examine existing employment practices and seek new opportunities to increase their participation rates. Notably, the newly introduced accreditation programme for health promotion practitioners in Australia provides a timely opportunity to consider the implications of credentialing—particularly whether this process will contribute to, or further impede, the empowerment of Aboriginal people in the health workforce.

Because the demand for AHWs far outstrips the supply (Gwynne and Lincoln, 2016) it makes little sense to restrict opportunities for Aboriginal people to these ‘official’ positions, particularly given the extensive research documenting the success of training local people to deliver quality and effective health promotion messages. Other employment and training models, like the ones we describe here, can provide community members with employment opportunities, pathways and skill development, impact positively on health outcomes for Aboriginal people and facilitate reconciliation in local communities (Cinelli and Peralta, 2015). But whether programmes adopt an AHW or an alternative employment model, clearly defining and conceptualizing the role of CHWs and the tasks they are meant to perform (as outlined in Table 1) will be critical in determining the kinds of employment, training and support necessary for success. As we previously discussed, ACCHOs are already engaging community members in non-AHW models. Documenting these experiences and models will also be critical if we are to develop knowledge about how best to ‘make space’ for Aboriginal people in the health workforce.

As the funding for the programmes we describe here only focused on uptake and effectiveness, we were unable to conduct an evaluation of our model in employing CHPs as the primary programme deliverers. Clearly, this is an area of future research and attention, as is the impact of CHP employment on both the workers and their broader communities. It was our experience that establishing CHP positions was both novel and challenging, and required ongoing ingenuity and perseverance. Continuous advocacy is needed to sustain this model, and we continue to seek the institutional-level change necessary for comprehensive reform. Importantly, the success of our programmes almost entirely hinges on the persistence and dedication of the SAPO involved and her ability to mentor CHPs while simultaneously advocating at the institutional level. Mentoring by the SAPO helped the CHPs to navigate the mainstream employment system and provided them with culturally safe support. The SAPO also acted as a link between CHPs and non-Aboriginal team members by providing valuable feedback and insights as to the additional support needed to assist CHPs, and about culturally appropriate adaptations to programme materials and training.

The SAPO’s role requires skills on many levels, including the development of relationships and trust with CHPs, and between non-Aboriginal partners. The enormous reliance on one person raises concerns about the appropriateness and sustainability of the current model as-is, while also underscoring the amount of workplace pressure placed on Aboriginal people to deliver outcomes in potentially unsupportive institutional environments. Ideally, large programmes involving multiple, geographically dispersed CHPs would be resourced sufficiently to reflect the demands on the time and the range of duties such roles often require and, therefore, to support multiple SAPOs as warranted. SAPOs also require support from senior levels of mainstream organizations to implement and support a new model of delivery.

As McPhail-Bell and colleagues (McPhail-Bell *et al.*, 2015) assert, ethical health promotion in Aboriginal communities requires that Aboriginal people are empowered to control the design of both health interventions and policies—which may include new models of employment and ownership of delivery—and not just be enlisted to deliver programmes developed elsewhere. Notably, our projects were externally developed and led by non-Aboriginal Australians. Shifting decision making to Aboriginal communities will require that non-Aboriginal professionals take a ‘backseat’ role, respect cultural differences and ways of doing things, be innovative, flexible and responsive in their approach, and support the CHPs in a discrete and collaborative manner.

Given the barriers we describe, ‘making space’ for CHP programmes and creating new CHP positions will require commitment, and should in no way minimize the importance and need for AHWs in health care. By distinguishing between types of CHW roles we aim to draw attention to other opportunities for Aboriginal ownership of health-promoting endeavours. The successful incorporation of CHPs and other kinds of Aboriginal CHW roles requires institutions to adapt to different models of mentoring, remuneration, training and support and to consider how these roles might be incorporated into already existing programmes. Closing gaps in disparities, requires new ways of thinking and acting and, therefore, a firm commitment to ensuring that health-promoting policies and programmes are culturally relevant and meaningful. To ensure true cultural relevance and create opportunities for Aboriginal people to design, lead and deliver health promotion programmes to their communities, such a commitment is not merely a preference but a requirement.

## ABOUT THE AUTHORS

NT is a Kamilaroi woman who partnered with JG (a non-Aboriginal woman) for more than 16 years to deliver community-based health promotion programmes in Australia. She was the SAPO described in this paper. KC is an American woman who has worked on CHW and lay educator programmes with First Nations people in the United States and Australia. CK and KG were collaborators in the implementation of the programmes described above. This paper came about when NT and JG shared with KC the barriers they experienced developing a non-AHW type position in the context of the programmes described herein. Together, we reflected on these experiences in the context of the broader international CHW literature to advance our thinking and articulation of the problems encountered. Our aim is to put forward an argument in support of action and research that exposes and addresses ongoing structural barriers to Indigenous community involvement in health promotion and health initiatives in Australian and beyond.
